# Why bad Moods Matter. William James on Melancholy, Mystic Emotion, and the Meaning of Life

**DOI:** 10.1007/s11406-017-9842-z

**Published:** 2017-05-09

**Authors:** Heleen Pott

**Affiliations:** 1 Department of Philosophy, Erasmus University Rotterdam, PO Box 1738, 3000 DR Rotterdam, Netherlands; 2 Zoutkeetsgracht 290, 1013 LC, Amsterdam, Netherlands

**Keywords:** James, Existential feelings, Moods, Phenomenology, Pragmatism

## Abstract

William James’s reputation in the field of emotion research is based on his early psychological writings where he defines emotions as ‘feelings of bodily changes’. In his later work, particularly in his study of mystic emotion (1902), James comes up with what looks like a completely different approach. Here his focus is on positive feelings of inspiration and joy, but also on downbeat moods like melancholy and depression. He examines how these feeling states give meaning to an individual’s life. Theorists often speculate about a gap between James’s early writings and his later work, and assume that the later James turned from an evolutionary-minded natural scientist into a metaphysical philosopher. In my paper, I follow Ratcliffe (2008) in his view that a sharply drawn line between the early and the late work is implausible and that James’s later study of mysticism fits nicely with his early psychology. Drawing on James (1902), I show how in his later work, James develops a theory of embodied emotions that anticipates the role ascribed by twentieth century phenomenology to anxiety and other ‘bad moods’, as possibilities for philosophical reflection and self-understanding.

## Introduction

For more than a century, the reputation of William James in the field of emotion research has been based on his article ‘What is an Emotion?’ in *Mind* (1884) and his chapter ‘The Emotions’ in *The Principles of Psychology* (1890), where he famously argued that emotions are ‘feelings of bodily changes’. During most of the twentieth century, James was criticized for reducing emotions to mere epiphenomena, resonances of physiological disturbances. In the last two decades, his view has been reassessed by ‘neo-Jamesians’ like Antonio Damasio and Jesse Prinz, who praise him for his focus on the adaptive (neuro)physiology of universal ‘basic emotions’.

Less widely known is that in later years, James continued his explorations in the affective domain, this time from a more phenomenological perspective. In *The Varieties of Religious Experience* (1902) he analyzes uniquely human feelings of mystical enthusiasm and joy, but also their opposites: feelings of disconnectedness, anxiety, melancholy, and nihilistic despair. His point of departure is that feelings associated with religion share a common psychological structure that can also be found in a more mundane context, for example in aesthetic emotions and drug experiences. He is particularly interested in how such ‘religious’ feelings give meaning to the life of individuals, and how they can be a cure for people suffering from melancholy and depression.

*Varieties* has hardly received any attention from psychologists working in the field of emotion – most likely because they mistook it for a work on theological issues. One notable exception is Averill (1992) who, in an interesting article titled ‘William James’s Other Theory of Emotion’, approves James’s later account for its focus on how emotions relate to creativity and the self, and advises the reader to throw away the early theory, as a reductive piece of hardcore scientism. The suggestion, shared by many of his biographers, is that James has made a radical turn in his thinking and by the end of his career gave up on his ‘tough minded’ naturalistic theory, in favor of a richer and more ‘tender minded’ philosophical outlook.

My goal here is to challenge this suggestion. In what follows I defend the idea that a sharply drawn line between James’s early approach of emotions as ‘feelings of bodily changes’ and his later explorations in the field of mystic emotion is implausible. I will go along with Ratcliffe (2005, 2008, 2010) and others in their view that the early James did not endorse the physiological James-Lange version of his theory, as is so often assumed in the textbooks. James was a dedicated scientist without ever becoming a materialist, a reductionist or a follower of British empiricism.

Much to the contrary, as a pioneer of American pragmatism, James was deeply committed to the investigation of consciousness as a first-person experience. Like other pragmatists, he adopted an evolutionary approach to the mind as an adaptive organ that selects for the organism’s survival salient features in the environment. Consciousness is not a passive mirror of nature; the function of the conscious mind is to actively keep the individual organism in tune with its ecological niche, and to structure behavior in such a manner that it conforms to the organism’s needs; first-person experiences are crucial for survival, according to James.

This Darwinian focus on consciousness as a first-person experience shows unexpected parallels with the project of phenomenology, a philosophical movement that emerged in Europe in the late nineteenth century. The similarities are so striking, that according to Linschoten (1968), Edie (1970), and many others, James could be considered to be a proto-phenomenologist. Interesting historical data to illustrate the link can be found in Spiegelberg (1965). During his years as a student in Europe, James became friends with the German psychologist and philosopher Carl Stumpf, who later would recommend James’ *Principles* to Husserl. The founder of phenomenology was deeply influenced by it and developed some of his ideas on intentionality by reading James. (Spiegelberg 1965, 11–116).

They differ however in that James’s view of consciousness is more embodied than Husserl’s; much like Merleau-Ponty half a century later, he takes the ‘lived body’ as point of departure for his phenomenology. The groundwork is already laid out in the *Principles*, where he defines psychology as ‘the science of mental life, both of its phenomena and their conditions’. (James 1890, I, 1). Clarifying how the first-person *phenomena* and the third-person physiological *conditions* of consciousness are correlated, is the aim of his new science. It is true that in his later work he gradually abandoned the physiological level of investigation; but the body was never far away in his work and even in his studies of mystic emotions in the *Varieties*, he makes it clear that it is through the body, that we feel connected with a universe divine.

Consequently, a re-evaluation of James’s early account of emotions as ‘feelings of bodily changes’ should start with a criticism of the dualism between mind and body, (cognitive) emotion and (non-cognitive) feeling, that prevents us to see the coherence in James’ work. What James meant with his statement was not that emotions are mere sensations inside the body, but that it is *through* the feeling body that we experience how the world matters to us. Feelings are cognitive, they are not just physiological states but they represent the significance of the world. In emotional feeling, body and world are inseparable, to experience one is to experience the other.

As Ratcliffe (2008) observes, the ‘coarse’ emotions in James’s early writings are just one out of many varieties of emotional experience that are examined in his work. Elsewhere he explores emotional feelings that are not directed to specific objects but are more like pre-intentional moods, dispositions, attitudes, or other background experiences. Ratcliffe speaks of ‘existential feelings’ (Ratcliffe 2008, 244): personal orientations through which experience as a whole is structured. Existential feelings constitute a sense of belonging, a sense of reality of self and of world; they are both ‘feelings of the body’ and ‘ways of finding oneself in the world’. (Ratcliffe 2008, 2).

In what follows I analyze the religious feelings in *Varieties* as a specific subclass of ‘existential feelings’. They are ‘more deep and more general’ (as James puts it) than other emotional feelings and figure prominently in first-person narratives of religious rapture and aesthetic experience, but also in testimonies of depression and other psychiatric illnesses. They can be found in religious and in non-religious persons alike. They differ from the standard emotions in James’s early work, in that they are not immediate responses to concrete objects in the world, but give shape and structure to the intentional ingredients of consciousness. In exceptional cases, they can be so overwhelming that they may effect in a radical transformation of the self and disclose a new world and a new way of living, constituted by new feelings of belonging, when the old world has become too difficult to act upon.

## Mystical Feelings in James’s *Varieties*

*Varieties* was published in 1902), eight years before James’s death, and five years before he published his major philosophical study *Pragmatism*. James (1981) It would become one of James’s most popular works. The book defends the controversial claim that in matters of religion, emotional experience has absolute priority over intellectual ideas. ‘I do believe that feeling is the deeper source of religion, and that philosophical and theological formulas are secondary products, like translations of a text into another tongue’, he writes (1902,337).

*Varieties* ignores everything that might interest theologians and directly focuses attention on the feelings associated with religious experience. James’ aim is to come up with some new ideas about their psychological function and meaning, particularly about the function of what he calls ‘mystical’ feelings: deep and intense feelings of connectedness to some unseen, higher reality – be it God, nature, or the world as a whole. According to James, mystical feelings are the essence of religion and their effect on the life of individuals is real and significant.

His phenomenological starting point is presented in the opening chapter, where he explicitly refutes ‘medical materialism’ and announces that he will explore religious and mystical experiences not as pathological physiological states, but as meaningful experiences that are continuous with other emotional phenomena. Object and context determine whether a feeling is religious or not, according to James. ‘(…) There is religious fear, religious love, religious awe (…). But religious love is only man’s natural emotion of love directed to a religious object (…); religious awe is the same organic thrill which we feel in a forest at twilight, or in a mountain gorge. Only this time it comes over us at the thought of our supernatural relations’(1902, 40).

James’ approach allows him to integrate his findings directly into a broader psychological framework that includes other emotions and feelings as well. The book draws on more than two hundred first-person narratives, written down by what James calls religious ‘geniuses’: celebrated saints and mystics, famous novelists, as well as ordinary people, who all documented their cosmic emotions in diaries, letters, and memoirs. The stories are examined by James from ‘a purely existential point of view’ (1902, 24): he delves into personal details, suggests psychological typologies, and theorizes about the meaning of mystic experience in the life of individuals.

The collection is broad: *Varieties* discusses not only feelings of religious rapture and awe that are related to traditions in Christianity, Islam, Buddhism, and Vedanta, but also more ordinary states of being aesthetically inspired by landscapes, poetry or music; all sorts of mood shifts and ‘psychedelic’ experiences induced by the use of alcohol, or drugs like laughing gas, peyote, chloroform, ether – in the psychedelic 1960s *Varieties* was something like a cult book; and finally a variety of feeling states such as existential ‘Angst’ and suicidal depression - experiences that mystics refer to as ‘the dark night of the soul’.

What they all share is a mystical quality, according to James. He defines mysticism as an alteration of consciousness, the opening of a window through which the soul ‘looks out upon a more extensive and inclusive world’ James (1902, 335). Most of the time, there is a sudden sense of mystical unity, an intuitive grasp of the ‘oneness’ of things; the experience is described as a heightened awareness of one’s environment, an insight into some deeper dimension of meaning not available to ordinary consciousness. ‘We pass into mystical states from out of ordinary consciousness as from a less into a more, as from a smallness into a vastness, and at the same time as from an unrest to a rest. We feel them as reconciling, unifying states. They appeal to the yes-function more than to the no-function in us’, he writes. (1902, 326).

This grasp of the ‘oneness’ of things characterizes all mystical states, from the lowest to the highest. Among the lower states are mystical conditions that nearly everyone has experienced at some time or another, feelings that are induced by chemical substances and bring a quick ‘high’, like temporary alterations of consciousness through alcohol: drunkenness is the great exciter of the *yes*-function in man, as James points out. Music is also placed on the mystical ladder, because ‘music gives us ontological messages which non-musical criticism is unable to contradict’ (1902,330). On a continuum, there are progressively greater feelings of joy, and enlargement of vision; the higher one climbs, the more intense are the positive feelings involved. At the top are full-fledged mystical states of ecstasy, experienced by only a small group of religious enthusiasts, who have thrown the doors of perception wide open and see the whole universe in a new light. Such full-blown mystical states are described as ‘(…) insights into depths of truth unplumbed by the discursive intellect. They are illuminations, revelations, full of significance and importance (…)’ (1902,300).

## Feelings as Ways of Worldmaking

In *Varieties*, the emphasis is on the ‘noetic’ quality of mystical experiences, on how they give access to a wider spiritual reality. Mystical feelings are experienced as states of knowledge that ‘(…) ‘break down the authority of the non-mystical or rationalistic consciousness, based upon the understanding and the senses alone. They show it to be only one kind of consciousness. They open out the possibility of other orders of truth’ (1902, 331). On the other hand, mystical knowledge is strictly intuitive and non-conceptual, it cannot be communicated.

This all goes well with the concept of ‘existential feelings’ (Ratcliffe 2005, 2008) as experiences that are grounding our sense of reality and belonging. As encompassing feelings they concern our relation to the world as a whole and are prior to any more specific relation to an object or situation; they are ‘possibility spaces’ (Ratcliffe 2010) that first make possible our grasp of objects in the world. The concept is inspired by Heidegger’s discussion of *Stimmungen* in *Being and Time* (1927) and in *The Fundamental Concepts of Metaphysics*, (1983) where he referred to this foundational layer of experience with the German word ‘Befindlichkeit’, to be translated as ‘a way of finding oneself in the world’, or ‘attunement’. *Stimmungen* determine how things matter to us, they constitute our pre-intentional openness to the world, according to Heidegger.

Ratcliffe (2008, 2010) points out that the category of existential feelings is broader than our ordinary concept of mood or *Stimmung*. He gives a list, including: ‘(…) feeling alive, dead, distant, dislodged, estranged, isolated, otherworldly, indifferent to everything, overwhelmed, suffocated, cut off, lost, disconnected, out of sorts, not oneself, out of touch with things, out of it, not quite with it, separate, in harmony with things, at peace with things…’. On the list are ‘(…) references to feelings of unreality, heightened existence, surreality, familiarity, unfamiliarity, strangeness, isolation, emptiness, belonging, being at home in the world, etc. (…)’ (Ratcliffe 2008, 68). The family includes forms of tacit understanding of our being in the world that may be so far beneath reflective awareness that they are more like dispositions; but also mood shifts that are so dramatic that they feel like an explosion of ecstasy, or a fit of despair; and extreme cases of ordinary emotion, like a fear so deep that the whole world seems threatening, or a joy so infinite that one feels on top of the world.

Understood as a subclass of existential feelings, mystical feelings differ from other feelings in that they are intensely felt changes in finding oneself in the world. At the same time, like all other existential feelings, they have the body as their sounding board - an aspect that is highlighted by James’s characterization of mystical feelings as experiences of intense pleasure and happiness, of reaching out and relaxation. First-hand reports of mystical experience describe a state of being overwhelmed and knocked over, of literally falling down and weeping of joy. Spiritual feelings are bodily feelings as well, as James observed already in his early work: ‘In all cases of intellectual or moral rapture we find that, unless there be coupled a bodily reverberation of some kind with the mere thought of the object and cognition of its quality (…), our state of mind can hardly be called emotional at all. (…) The bodily sounding board is at work, as careful introspection will show, far more than we usually suppose.’ (James 1890, 470–1).

The role of the body as a sounding board also explains the overwhelming sense of reality that characterizes mystical feeling. Mystical experiences feel intensely real, they are authoritative over the individuals to whom they come. ‘For those who have them, mystical experiences are as direct perceptions of fact’, James writes; ‘…that is, they are face to face presentations of what seems immediately to exist.’ (1902, 332). *Varieties* leaves it an open question whether or not there really exists ‘something there’, some divine entity that transcends the natural world and manifests itself in mystic emotion. James is intentionally agnostic on this issue. From a phenomenological perspective, he is not interested to investigate the *origins* of religious emotion, he just aims to give a careful description of the *experience* and to clarify its meaning as a safety net in the personal struggle with depression and the loss of meaning in the modern world. Besides, this phenomenological methodology can be easily complemented with the pluralistic idea that multiple worlds are possible and that there may be more to reality than our natural world – a ‘more’ that generates positive effects from which ordinary experience is shut off. Although this ‘more’ cannot be objectively justified, the changes, conversions, and self-transformations induced by it are real, and accompanied by feelings of immense joy, as James observes. ‘Happiness! Religion is one of the ways in which men gain this gift. Easily, permanently, and successfully, it often transforms the most intolerable misery into the profoundest and most enduring happiness.’ (1902,150).

Therefore, he sees religion as a helpful natural faculty. In the absence of any objective proof for the divine object of religious experience, ‘(….) if the fruits for life of the state of conversion are good, we ought to idealize it and venerate it, even though it be a piece of natural psychology.’ (1902, 195).

## Melancholy and the ‘Sick Soul’

Although James sympathized with religious believers, he was not a positive believer himself. ‘My personal position is simple’, he says in a letter to Henry Leuba (14 April 1904): ‘I have no living sense of commerce with a God (…). I envy those who have, for I know that the addition of such a sense would help me greatly. (…) Now, although I am devoid of *Gottesbewustsein*, there is something in me which makes response when I hear utterances from that quarter, I recognize the deeper voice…Call this my mystical germ.’

James’s ‘mystical germ’ was intimately connected to his melancholic constitution. In his twenties he went through experiences of melancholy and depression, and all his life he was in the grip of a pessimistic mindset that he himself called the illness of the ‘sick soul’. He was fully aware that mystical emotion may mysteriously create new centers of personal energy; therefore, he always silently hoped that a God would speak to him, in some dark night. In *Varieties*, he examines experiences of melancholy, anxiety, and depression more in detail. James has his own emotional symptoms in mind when he describes a ‘worst kind of melancholy’, in the guise of a memoir given to him by an anonymous Frenchman:

‘I went one evening into a dressing room in the twilight to procure some article that was there; when suddenly there fell upon me without warning, just as if it came out of the darkness, a horrible fear of my own existence. Simultaneously there arose in my mind the image of an epileptic patient whom I had seen in the asylum, a black-haired youth, with greenish skin, entirely idiotic, who used to sit all day on one of the benches, or rather shelves against the wall, with his knees drawn up against his chin. That shape am I, I felt, potentially. There was such a horror, …that it was as if something solid within my breast gave way entirely…and I became a mass of quivering fear. After this, the universe had changed for me altogether. I awoke morning after morning with a horrible dread at the pit of my stomach, and with a sense of the insecurity of life that I never knew before, and that I have never felt since.’

That evening everything changes dramatically for James, all everyday familiarity collapses, his confidence in life is gone. A deep anxiety that human life is meaningless makes him see his place in the world in a completely different light. He concludes his report with the intriguing claim that the experience has ‘religious bearing’ and is similar to religious experiences, but ‘more by contrast than identity with them’. ‘I mean that the fear was so invasive and powerful that if I had not clung to scripture-like texts like: “the eternal God is my refuge…”, I think I would have grown really insane.’

This ‘reverse religious experience’ in which the world becomes completely *unheimisch* fits neatly with one of the central psychological distinctions in *Varieties*: the distinction between the healthy minded individual and the ‘sick soul’. Healthy minded people are natural born optimists, they find life easy and have a deep sense of the goodness of the universe, against all better evidence. Sick souls however, find that ‘(…) unexpectedly, from the bottom of every fountain of pleasure, something bitter rises up: a touch of nausea, …a whiff of melancholy, … a feeling coming from a deeper region and often with an appalling convincingness’. (1902, 120)

On the basis of these dispositions James lays out the scope of a phenomenological psychology, grounded in two modes of feeling that are so foundational that they come close to personality types. They determine two distinct kinds of openness to the world: on the one hand a sense of belonging, a feeling of being safe and at home in the world; on the other hand a sense of being disconnected, of loneliness and feeling insecure. Healthy-minded individuals are positively attuned to life, they seem to be blind for all the negative aspects, they accept suffering and death as unproblematic and never lose their optimistic attitude. The sick soul however feels anxious and isolated, (s)he is a natural born pessimist.

For biographical but also for philosophical reasons, James’ sympathy is with the ‘sick soul’, who in his view has a deeper, more realistic sense of life than his optimistic counterpart. It is the sick soul who may experience profound shifts in how he or she feels attuned to the world; shifts that give a lot of insight into the foundational meaning structures of human life. James’ favorite example of the ‘sick soul’ is inspired by Tolstoy’s (1882) autobiographical *A Confession*, where the author describes an experience of severe depression and how he was saved from it by mystical conversion.

Tolstoy observes how one day, he cannot enjoy the ordinary pleasures of living any longer. All activity becomes meaningless, nothing matters any more. Obsessed with the idea that suffering and death are inevitable, and that ultimately all human effort is futile, he sees no reason why he wouldn’t kill himself. ‘I felt’, writes Tolstoy, that something had broken within me on which my life had always rested, that I had nothing left to hold on to, and that morally my life had stopped. An invincible force impelled me to kill myself, in one way or another.’ (Tolstoy, in James 1902, 133) He feels as if he is at the bottom of a dark pit. The world has turned into an evil place in which he can never be at home anymore.

What saves Tolstoy from suicidal despair is a mysterious religious awakening, accompanied by a sudden explosion of positive energy, and the overwhelming sense that all is ultimately well, even though the outer conditions have remained the same. Afterwards, he feels like reborn; the world has regained its color and meaning. He returns to the simple faith of the Russian peasants and completely changes his way of life.

Severe depression, as in the case of Tolstoy, is an existential condition that blocks one’s possibilities to find meaning in the world, and to be comfortable together with others. It is experienced as a loss of appetite for all activity, a complete absence of pleasure in anything. It feels like drowning in a pool of misery. In Tolstoy’s case, the therapy of depression exists in a completely unexpected religious experience, a mystical conversion by which his soul is healed and Tolstoy himself becomes a new person. ‘The process is one of redemption, n ot of mere reversion to natural health, and the sufferer, when saved, is saved by what seems to him a second birth, a deeper kind of conscious being than he could enjoy before’, writes James (1902, 135). The whole experience shows a striking similarity with Sartre’s famous description of emotion as a ‘magical transformation of the world’ (Sartre 1939), where the person symbolically confers qualities on the world that cannot be given deterministically, when the paths in the world are blocked but action must go forward. The transformation results in a reorientation that may alter one’s self-image and the structure of one’s relationship with reality completely, in a manner that is experienced as positive.

James was fascinated by the idea of conversion as a magical cure for the depressed individual*.* He interpreted the pessimistic mindset of the ‘sick soul’ as a religious disease that paralyzes all action impulses and alienates the self from the world of everyday life. Recovery from this disease is experienced as a personal regeneration, and narrated most of the time in specifically religious terms. ‘To be converted, to be regenerated, to receive grace, …are so many phrases which denote the process, gradual or sudden, by which a self hitherto divided, and consciously wrong, inferior, and unhappy, becomes unified and consciously right, superior and happy, in consequence of its former hold upon religious realities’, he writes (1902, 160).

Therefore, although the special manifestations of religion may be self-deceptive, James holds that the life of religion as a whole is among mankind’s most important biological functions.

## Why bad Moods Matter

This particular interest in mystic emotion as a way of making the world new, explains why James gave so much attention to what he called religious ‘geniuses’, who ‘(…) have been creatures of exalted emotional sensibility. Often they have led a discordant inner life, and suffered from melancholy during a part of their career. They have known no measure, been liable to obsessions and fixed ideas; and frequently, they have fallen into trances, heard voices, seen visions, and presented peculiarities which are ordinarily classed as pathological’ (1902, 25).

The stories told by these geniuses are not about religious confidence, but about melancholy and depression, and about dramatic mood shifts that sometimes come close to psychiatric illness. James, however, is not interested in the pathological origins of mystic emotions but in their illustrative value. What these limit cases provide him with are dramatic changes in the sense of belonging, that give a better understanding of the deep structures of human experience and consciousness itself. ‘I say (…) that it always leads to a better understanding of a thing’s significance to consider its exaggerations and perversions, its equivalents and substitutes, and nearest relatives elsewhere (…) Insane conditions have this advantage, that they isolate special factors of mental life, and enable us to inspect them unmasked by their more usual surroundings.’ (1902, 35–36).

Deep negative moods are much more unsettling in their effect on our perceptions, thoughts, feelings, and our overall state of mind than positive moods. They obstruct habitual ways of thinking and acting in the world, and shake us out of our comfortable position. There is a lonesome quality to bad moods. In a state of anxiety one feels uncanny, thrown back on oneself; entities within the normal world become indifferent, everything sinks away, and there is no way back to the comfort of social life anymore. Existential phenomenologists have pointed out that such experiences of disconnectedness are an opportunity to catch a deeper, more philosophical understanding of the very nature of a person’s being in the world – which is precisely why they were referred to by Heidegger as *Grundstimmungen.*

Like Heidegger, James considers negative feelings such as deep anxiety or Angst to be more ‘metaphysical’ than most other feelings. Angst makes us aware of inner tensions in our self- understanding, as he illustrates in the *Varieties,* memorizing his own experience of existential panic in the asylum where anxiety took the form of a nightmarish delusion, similar to seeing a demonic ghost that kept haunting him for months, maybe even years. What dawns in this ‘reverse religious experience’, as James called it, is the possibility that reason and truth are ultimately without foundation, that human life is nasty, brutish, and short; and there is no escape: ‘…..that shape am I’. Angst discloses the tragic character of human experience itself, in a naturalistic universe where God is dead or silent.

Other negative conditions like melancholy and depression may induce fundamental reflection as well; they make us ask questions that in normal circumstances would never be asked. In *A Confession*, Tolstoy writes: ‘Something very strange began to happen to me. At first I experienced moments of perplexity (…) as though I did not know how to live. ..They were always expressed by the questions: Why? What is it for? What does it lead to?’ (Tolstoy, in James 1902, 132). Tolstoy had no answer, because traditional answers like family, work, love, art, no longer struck him as adequate. What his depressed mindset disclosed and what remains hidden in more ordinary feeling states is that human life needs a certain amount of ‘intoxication’, in order to function smoothly. As Tolstoy puts it: ‘One can live only so long as one is drunk with life; but when one grows sober, one cannot fail to see that it is all a stupid cheat’ (Tolstoy, in James 1902, 132). Tolstoy’s sober truth is that all human activity will ultimately leave no trace behind, and that this is an unbearable reality to live with. Humans are metaphysical animals, they need a positive horizon, a vision of hope.

For James, a positive horizon is precisely what religious emotions can provide us with. In the course of the evolutionary-biological development they have evolved as important ingredients of a successful coping strategy in times of distress. Living in the age of science and modernity makes us all prone to become sick souls, in James’s view. And the world picture of scientific materialism only adds to sadness and pessimism, it can offer us nothing but the perspective of meaninglessness and the death of the planet as ultimate horizon. As he puts it: ‘For naturalism, mankind is in a position similar to that of a set of people living on a frozen lake, surrounded by cliffs over which there is no escape, yet knowing that little by little the ice is melting, and the inevitable day drawing near when the last film of it will disappear, and to be drowned ignominiously will be the human creature’s portion. The merrier the skating, the warmer and more sparkling the sun by day, and the ruddier the bonfires by night, the more poignant the sadness with which one must take the meaning of the total situation.’ (1902, 124).

Mystical experience presents a vision of the universe that is in harmony with our human need for connectedness. It cannot offer a proof for God or eternal life, but at least allows for the possibility of a more meaningful reality that may be hidden from everyday consciousness. Therefore, James has never the intention to debunk mystical engagements as mental illness or mood disorder. Much to the contrary, being a pragmatist, he takes the possible truth of supernatural orders quite seriously: they may be real since they produce real effects. In so far as mystic emotions are successful coping mechanisms that help individual humans effectively deal with negative mindsets, they open out the possibility of deeper dimensions of truth ‘in which as far as anything in us vitally responds to them, we may freely continue to have faith’ (1902, 331).

In *Pragmatism*, published a few years later, James elaborated on this position and defined a pragmatist as someone who is willing to live on a scheme of uncertified possibilities, which he feels he can trust.

## Conclusion

In *Varieties,* James explores a range of existential moods and feelings that are traditionally understood in religious terms but are continuous with other emotions. He gives special attention to the subclass of ‘mystic’ emotion: experiences through which the mind ‘looks out upon a more extensive and inclusive world’ (1902, 335).

James’s study of mystic emotion complements his early psychological work on object-directed standard emotions like fear, sadness and anger. The common characteristics are, first, that standard emotions as well as mystical emotions are described as personal experiences that have the body as their sounding board – both are experiences of meaning through the body; second, that standard emotions and mystical emotions are both seen as crucial to motivation and presented as ways of coping with difficult situations in life – standard emotions deal with events and objects in the world outside, mystical emotions deal with situations in the inner world, the world of personal ideas, values, and world views.

With his analysis of Angst as a feeling state that induces philosophical self-understanding, James anticipates a central theme in existential phenomenology. With his Darwinian account of mystic emotion as a way of world making, he reveals a pragmatic ontology avant la lettre.

In *Varieties*, the two philosophical positions, existential phenomenology and pragmatism, elegantly converge into what can be seen as the first phenomenological psychology of embodied emotions in Western history.
